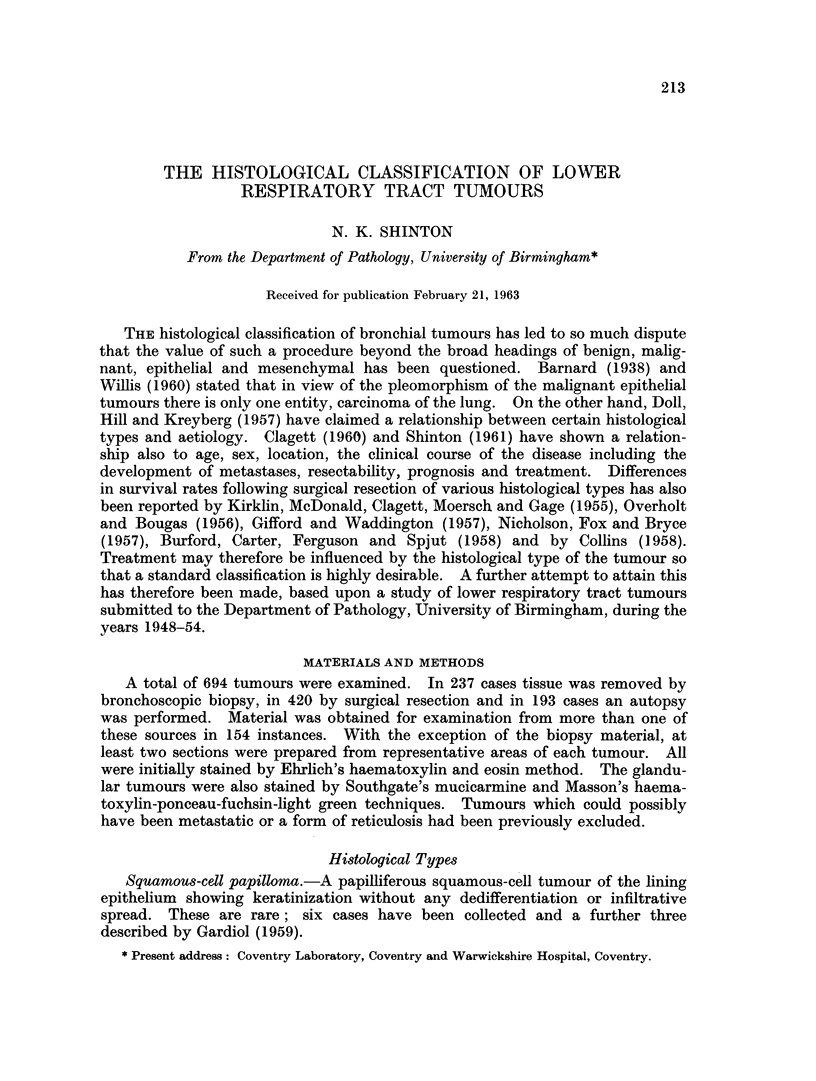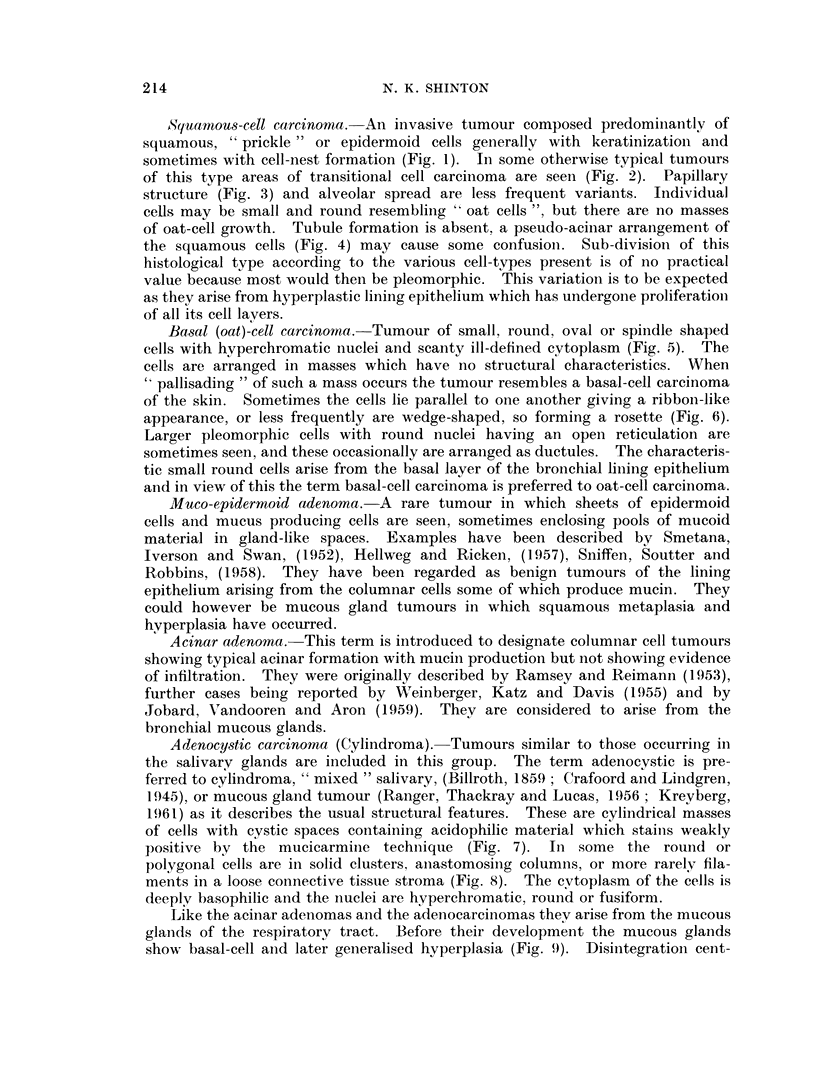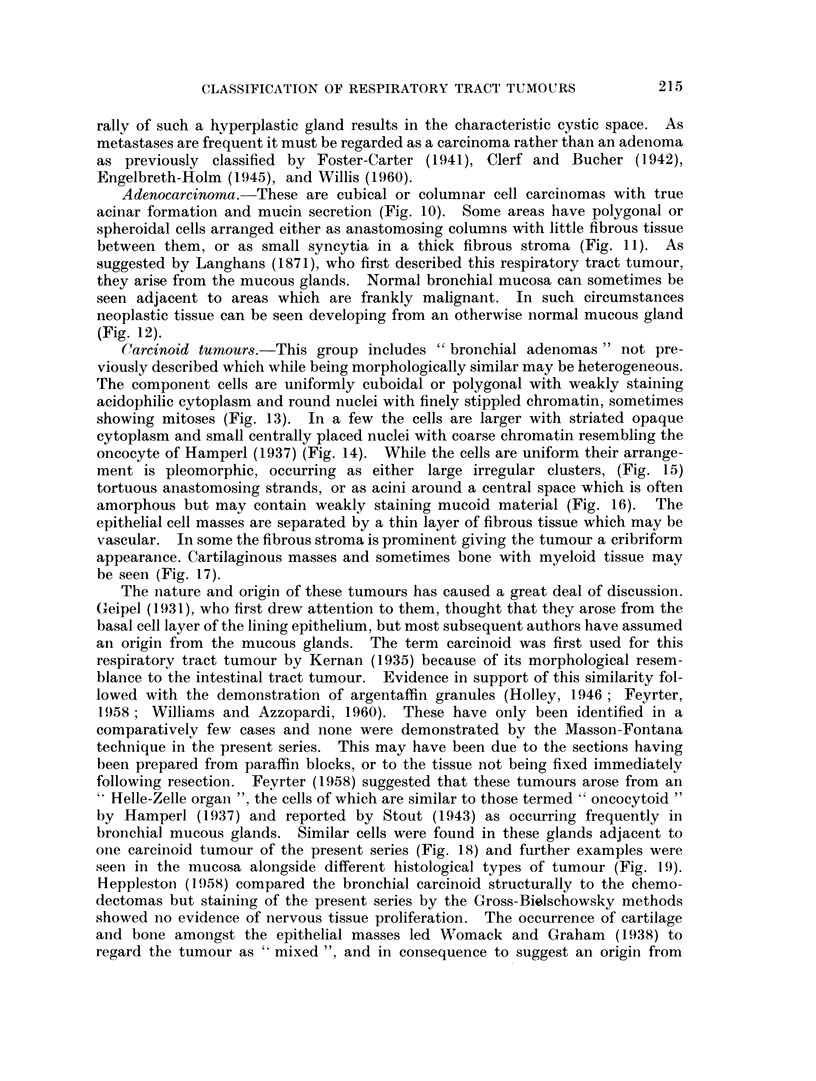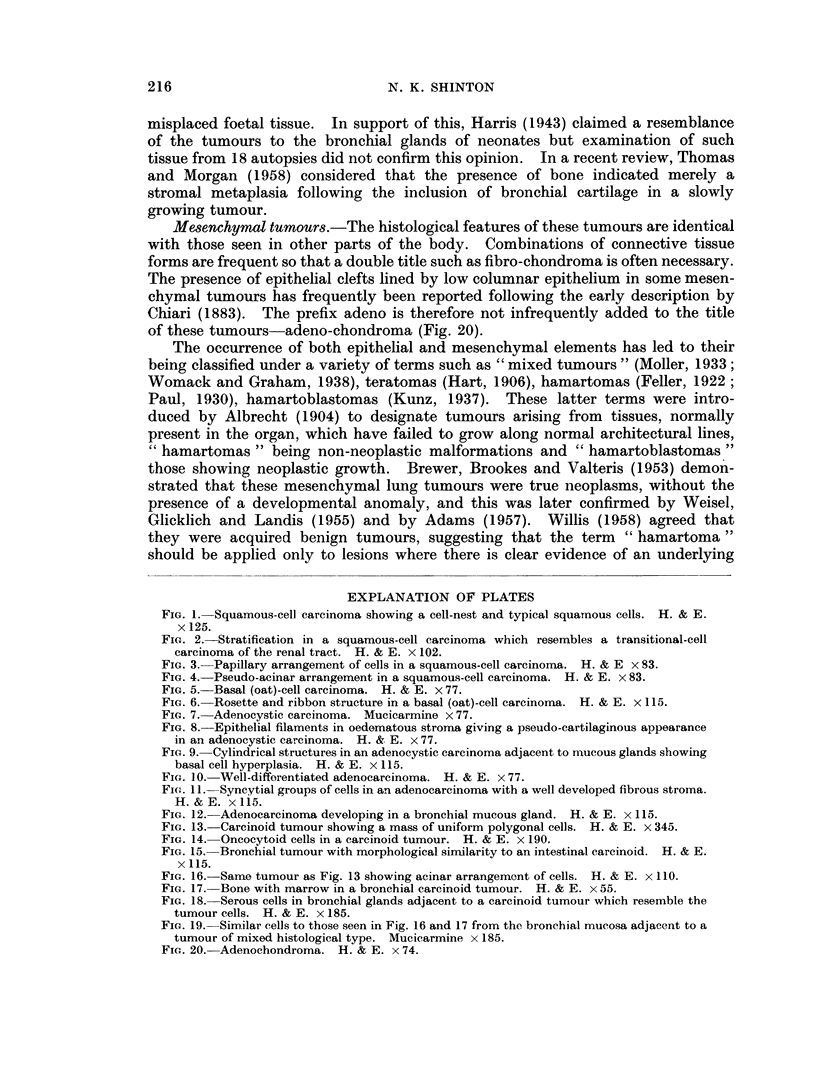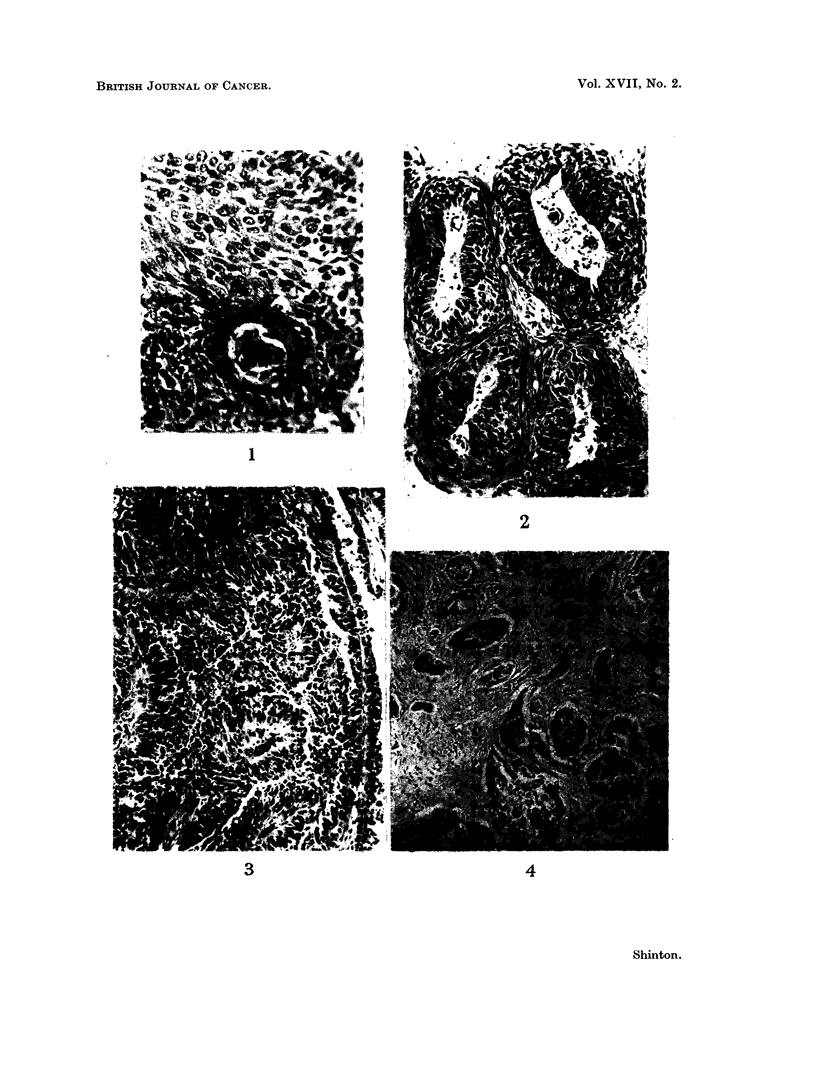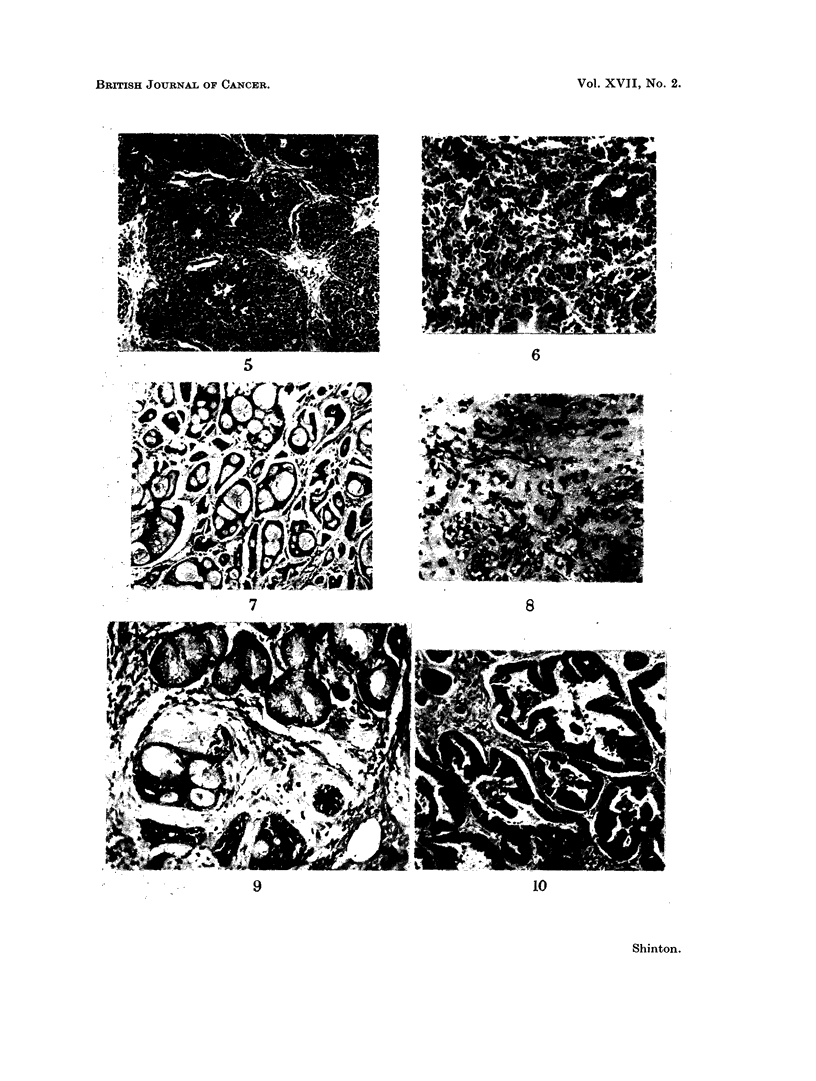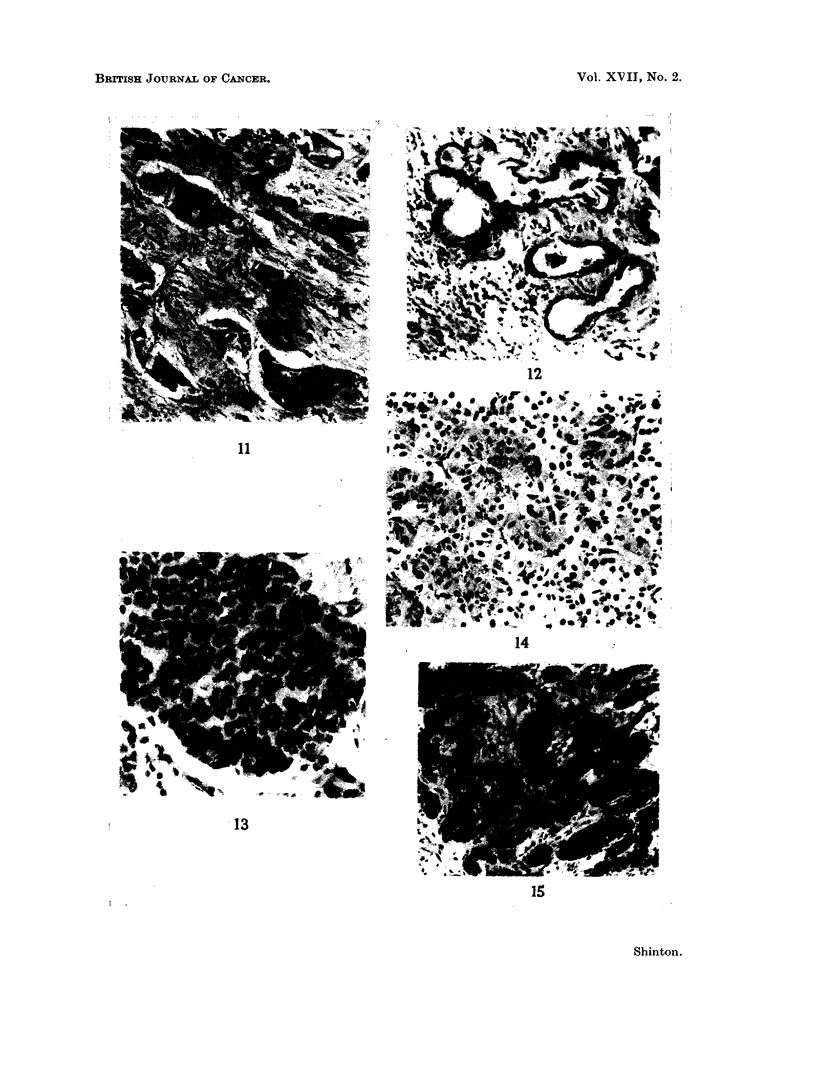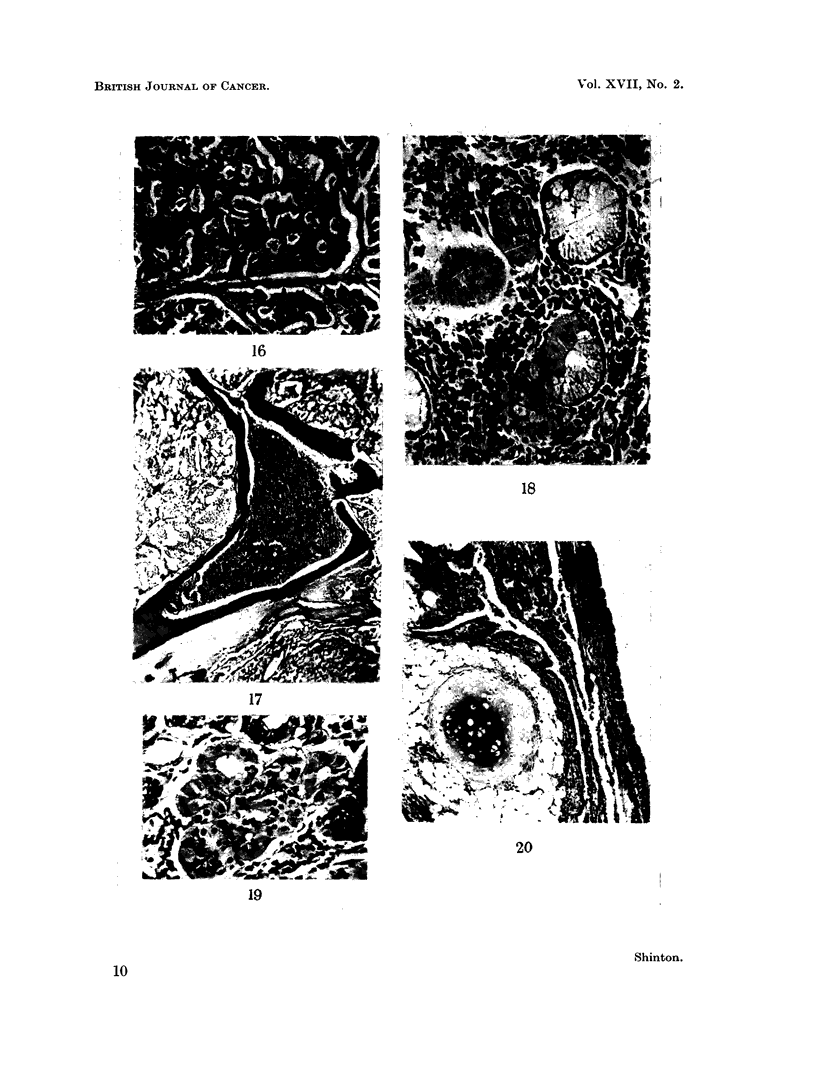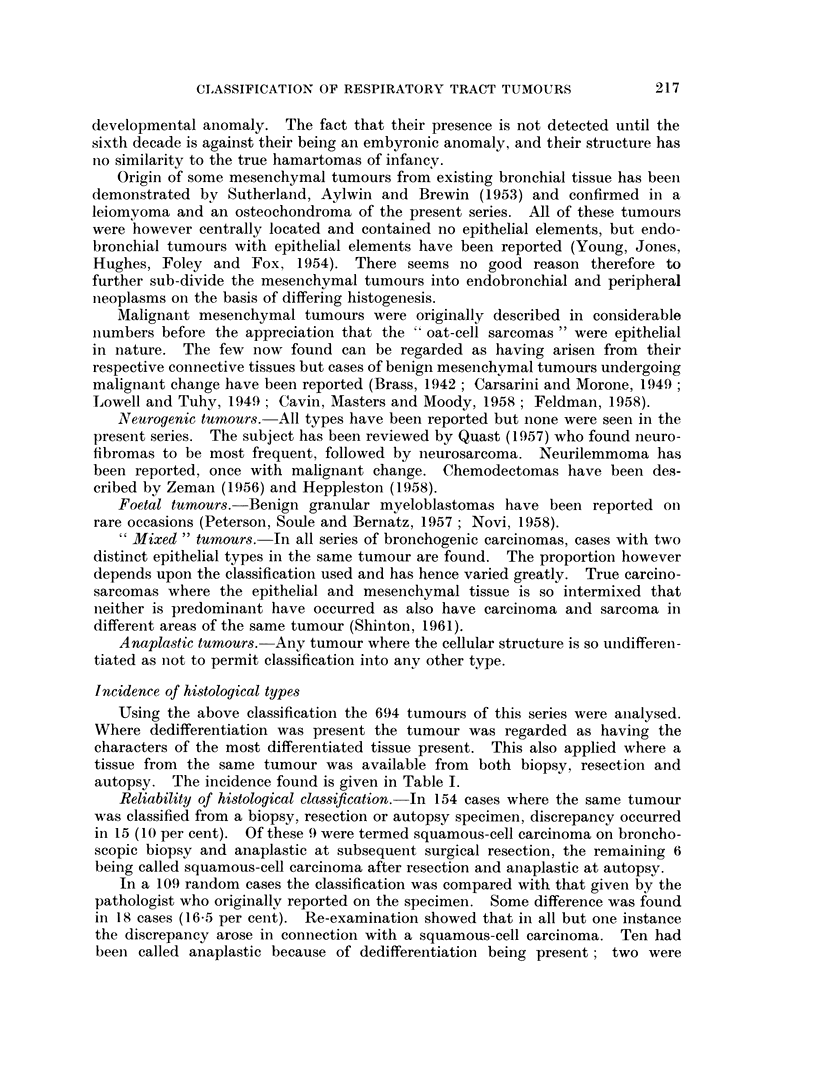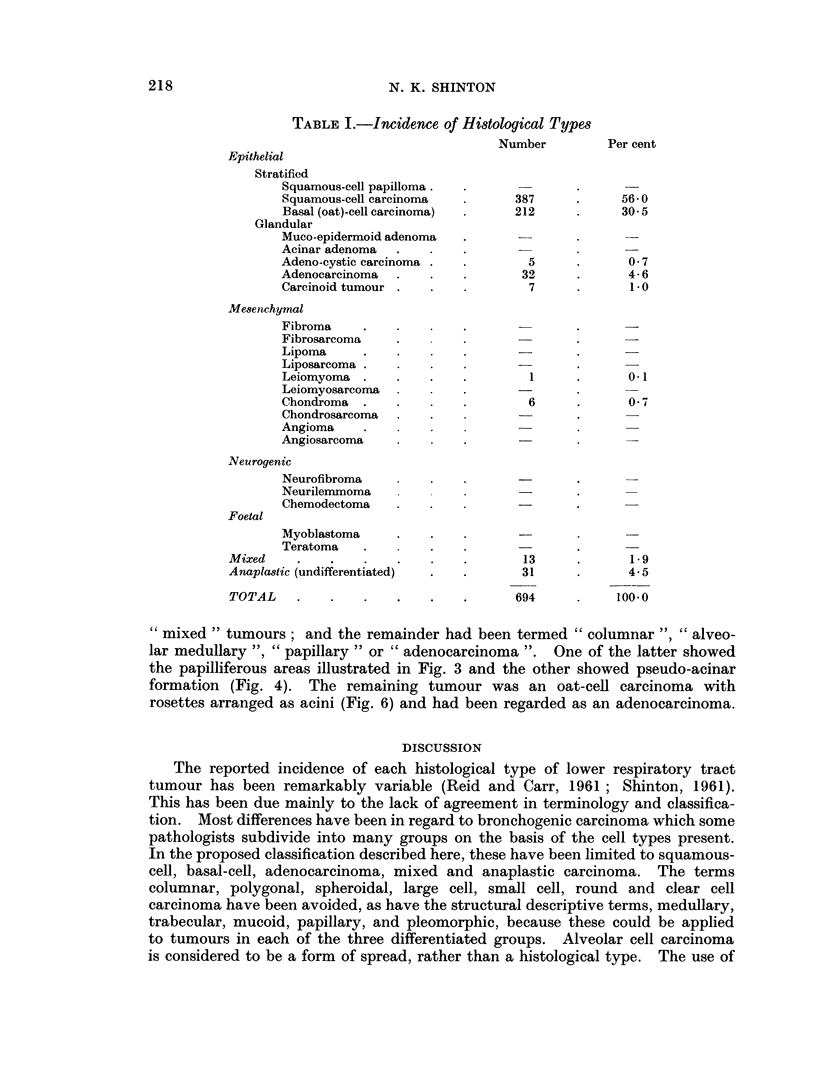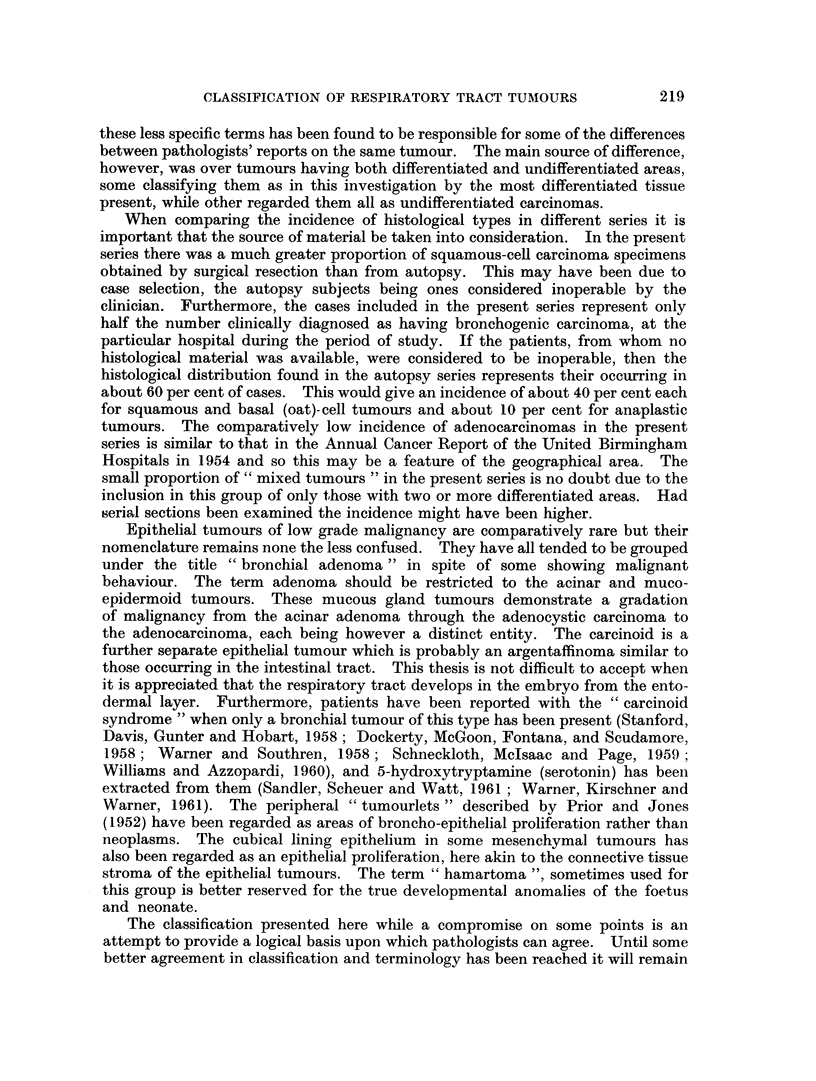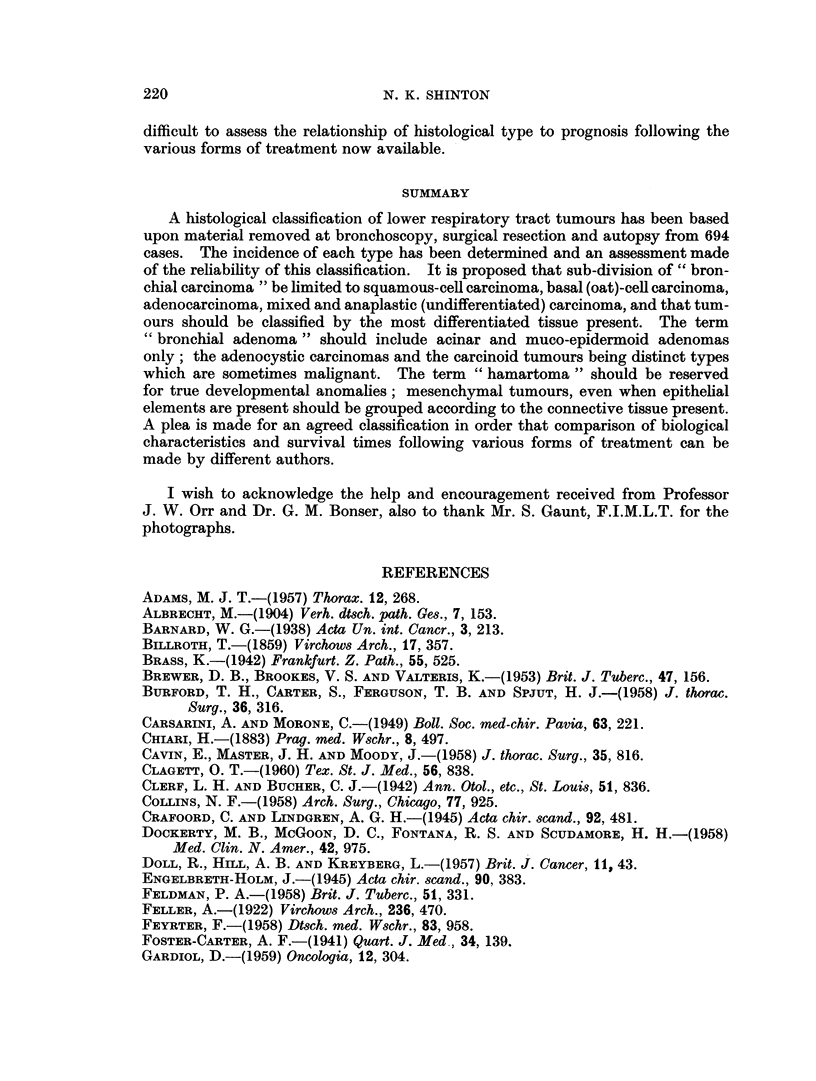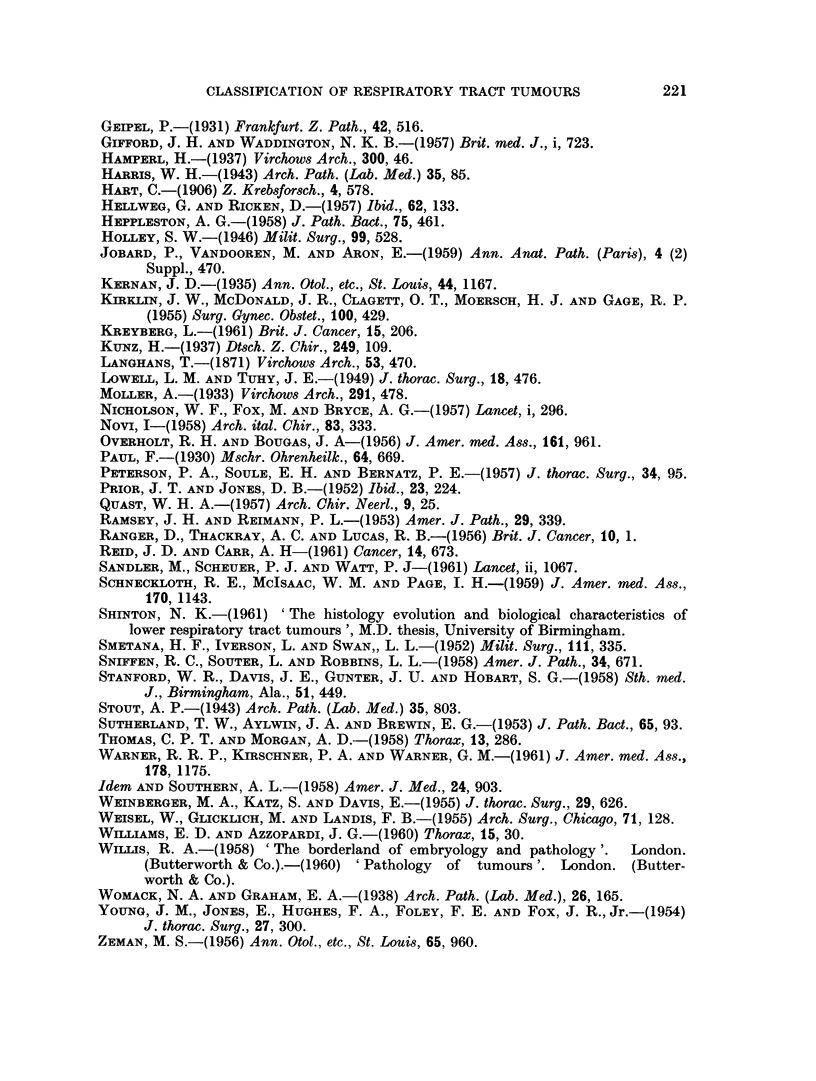# The Histological Classification of Lower Respiratory Tract Tumours

**DOI:** 10.1038/bjc.1963.30

**Published:** 1963-06

**Authors:** N. K. Shinton

## Abstract

**Images:**


### THE HISTOLOGICAL CLASSIFICATION OF LOWER RESPIRATORY TRACT TUMOURS

N. K. SHINTON

*From the Department of Pathology, University of Birmingham**

Received for publication February 21, 1963

THE histological classification of bronchial tumours has led to so much dispute that the value of such a procedure beyond the broad headings of benign, malignant, epithelial and mesenchymal has been questioned. Barnard (1938) and Willis (1960) stated that in view of the pleomorphism of the malignant epithelial tumours there is only one entity, carcinoma of the lung. On the other hand, Doll, Hill and Kreyberg (1957) have claimed a relationship between certain histological types and aetiology. Clagett (1960) and Shinton (1961) have shown a relationship also to age, sex, location, the clinical course of the disease including the development of metastases, resectability, prognosis and treatment. Differences in survival rates following surgical resection of various histological types has also been reported by Kirklin, McDonald, Clagett, Moersch and Gage (1955), Overholt and Bougas (1956), Gifford and Waddington (1957), Nicholson, Fox and Bryce (1957), Burford, Carter, Ferguson and Spjut (1958) and by Collins (1958). Treatment may therefore be influenced by the histological type of the tumour so that a standard classification is highly desirable. A further attempt to attain this has therefore been made, based upon a study of lower respiratory tract tumours submitted to the Department of Pathology, University of Birmingham, during the years 1948–54.

### MATERIALS AND METHODS

A total of 694 tumours were examined. In 237 cases tissue was removed by bronchoscopic biopsy, in 420 by surgical resection and in 193 cases an autopsy was performed. Material was obtained for examination from more than one of these sources in 154 instances. With the exception of the biopsy material, at least two sections were prepared from representative areas of each tumour. All were initially stained by Ehrlich's haematoxylin and eosin method. The glandular tumours were also stained by Southgate's mucicarmine and Masson's haematoxylin-ponceau-fuchsin-light green techniques. Tumours which could possibly have been metastatic or a form of reticulosis had been previously excluded.

### *Histological Types*

*Squamous-cell papilloma.*—A papilliferous squamous-cell tumour of the lining epithelium showing keratinization without any dedifferentiation or infiltrative spread. These are rare; six cases have been collected and a further three described by Gardiol (1959).

* Present address: Coventry Laboratory, Coventry and Warwickshire Hospital, Coventry.

*Squamous-cell carcinoma.*—An invasive tumour composed predominantly of squamous, " prickle " or epidermoid cells generally with keratinization and sometimes with cell-nest formation (Fig. 1). In some otherwise typical tumours of this type areas of transitional cell carcinoma are seen (Fig. 2). Papillary structure (Fig. 3) and alveolar spread are less frequent variants. Individual cells may be small and round resembling " oat cells ", but there are no masses of oat-cell growth. Tubule formation is absent, a pseudo-acinar arrangement of the squamous cells (Fig. 4) may cause some confusion. Sub-division of this histological type according to the various cell-types present is of no practical value because most would then be pleomorphic. This variation is to be expected as they arise from hyperplastic lining epithelium which has undergone proliferation of all its cell layers.

*Basal (oat)-cell carcinoma.*—Tumour of small, round, oval or spindle shaped cells with hyperchromatic nuclei and scanty ill-defined cytoplasm (Fig. 5). The cells are arranged in masses which have no structural characteristics. When " pallisading " of such a mass occurs the tumour resembles a basal-cell carcinoma of the skin. Sometimes the cells lie parallel to one another giving a ribbon-like appearance, or less frequently are wedge-shaped, so forming a rosette (Fig. 6). Larger pleomorphic cells with round nuclei having an open reticulation are sometimes seen, and these occasionally are arranged as ductules. The characteristic small round cells arise from the basal layer of the bronchial lining epithelium and in view of this the term basal-cell carcinoma is preferred to oat-cell carcinoma.

*Muco-epidermoid adenoma.*—A rare tumour in which sheets of epidermoid cells and mucus producing cells are seen, sometimes enclosing pools of mucoid material in gland-like spaces. Examples have been described by Smetana, Iverson and Swan, (1952), Hellweg and Ricken, (1957), Sniffen, Soutter and Robbins, (1958). They have been regarded as benign tumours of the lining epithelium arising from the columnar cells some of which produce mucin. They could however be mucous gland tumours in which squamous metaplasia and hyperplasia have occurred.

*Acinar adenoma.*—This term is introduced to designate columnar cell tumours showing typical acinar formation with mucin production but not showing evidence of infiltration. They were originally described by Ramsey and Reimann (1953), further cases being reported by Weinberger, Katz and Davis (1955) and by Jobard, Vandooren and Aron (1959). They are considered to arise from the bronchial mucous glands.

*Adenocystic carcinoma* (Cylindroma).—Tumours similar to those occurring in the salivary glands are included in this group. The term adenocystic is preferred to cylindroma, " mixed " salivary, (Billroth, 1859 ; Crafoord and Lindgren, 1945), or mucous gland tumour (Ranger, Thackray and Lucas, 1956 ; Kreyberg, 1961) as it describes the usual structural features. These are cylindrical masses of cells with cystic spaces containing acidophilic material which stains weakly positive by the mucicarmine technique (Fig. 7). In some the round or polygonal cells are in solid clusters, anastomosing columns, or more rarely filaments in a loose connective tissue stroma (Fig. 8). The cytoplasm of the cells is deeply basophilic and the nuclei are hyperchromatic, round or fusiform.

Like the acinar adenomas and the adenocarcinomas they arise from the mucous glands of the respiratory tract. Before their development the mucous glands show basal-cell and later generalised hyperplasia (Fig. 9). Disintegration centrally of such a hyperplastic gland results in the characteristic cystic space. As metastases are frequent it must be regarded as a carcinoma rather than an adenoma as previously classified by Foster-Carter (1941), Clerf and Bucher (1942), Engelbreth-Holm (1945), and Willis (1960).

*Adenocarcinoma.*—These are cubical or columnar cell carcinomas with true acinar formation and mucin secretion (Fig. 10). Some areas have polygonal or spheroidal cells arranged either as anastomosing columns with little fibrous tissue between them, or as small syncytia in a thick fibrous stroma (Fig. 11). As suggested by Langhans (1871), who first described this respiratory tract tumour, they arise from the mucous glands. Normal bronchial mucosa can sometimes be seen adjacent to areas which are frankly malignant. In such circumstances neoplastic tissue can be seen developing from an otherwise normal mucous gland (Fig. 12).

*Carcinoid tumours.*—This group includes " bronchial adenomas " not previously described which while being morphologically similar may be heterogeneous. The component cells are uniformly cuboidal or polygonal with weakly staining acidophilic cytoplasm and round nuclei with finely stippled chromatin, sometimes showing mitoses (Fig. 13). In a few the cells are larger with striated opaque cytoplasm and small centrally placed nuclei with coarse chromatin resembling the oncocyte of Hamperl (1937) (Fig. 14). While the cells are uniform their arrangement is pleomorphic, occurring as either large irregular clusters, (Fig. 15) tortuous anastomosing strands, or as acini around a central space which is often amorphous but may contain weakly staining mucoid material (Fig. 16). The epithelial cell masses are separated by a thin layer of fibrous tissue which may be vascular. In some the fibrous stroma is prominent giving the tumour a cribriform appearance. Cartilaginous masses and sometimes bone with myeloid tissue may be seen (Fig. 17).

The nature and origin of these tumours has caused a great deal of discussion. Geipel (1931), who first drew attention to them, thought that they arose from the basal cell layer of the lining epithelium, but most subsequent authors have assumed an origin from the mucous glands. The term carcinoid was first used for this respiratory tract tumour by Kernan (1935) because of its morphological resemblance to the intestinal tract tumour. Evidence in support of this similarity followed with the demonstration of argentaffin granules (Holley, 1946 ; Feyrter, 1958 ; Williams and Azzopardi, 1960). These have only been identified in a comparatively few cases and none were demonstrated by the Masson-Fontana technique in the present series. This may have been due to the sections having been prepared from paraffin blocks, or to the tissue not being fixed immediately following resection. Feyrter (1958) suggested that these tumours arose from an " Helle-Zelle organ ", the cells of which are similar to those termed " oncocytoid " by Hamperl (1937) and reported by Stout (1943) as occurring frequently in bronchial mucous glands. Similar cells were found in these glands adjacent to one carcinoid tumour of the present series (Fig. 18) and further examples were seen in the mucosa alongside different histological types of tumour (Fig. 19). Heppleston (1958) compared the bronchial carcinoid structurally to the chemodectomas but staining of the present series by the Gross-Bielschowsky methods showed no evidence of nervous tissue proliferation. The occurrence of cartilage and bone amongst the epithelial masses led Womack and Graham (1938) to regard the tumour as " mixed ", and in consequence to suggest an origin from misplaced foetal tissue. In support of this, Harris (1943) claimed a resemblance of the tumours to the bronchial glands of neonates but examination of such tissue from 18 autopsies did not confirm this opinion. In a recent review, Thomas and Morgan (1958) considered that the presence of bone indicated merely a stromal metaplasia following the inclusion of bronchial cartilage in a slowly growing tumour.

*Mesenchymal tumours.*—The histological features of these tumours are identical with those seen in other parts of the body. Combinations of connective tissue forms are frequent so that a double title such as fibro-chondroma is often necessary. The presence of epithelial clefts lined by low columnar epithelium in some mesenchymal tumours has frequently been reported following the early description by Chiari (1883). The prefix adeno is therefore not infrequently added to the title of these tumours—adeno-chondroma (Fig. 20).

The occurrence of both epithelial and mesenchymal elements has led to their being classified under a variety of terms such as " mixed tumours " (Moller, 1933; Womack and Graham, 1938), teratomas (Hart, 1906), hamartomas (Feller, 1922; Paul, 1930), hamartoblastomas (Kunz, 1937). These latter terms were introduced by Albrecht (1904) to designate tumours arising from tissues, normally present in the organ, which have failed to grow along normal architectural lines, " hamartomas " being non-neoplastic malformations and " hamartoblastomas " those showing neoplastic growth. Brewer, Brookes and Valteris (1953) demonstrated that these mesenchymal lung tumours were true neoplasms, without the presence of a developmental anomaly, and this was later confirmed by Weisel, Glicklich and Landis (1955) and by Adams (1957). Willis (1958) agreed that they were acquired benign tumours, suggesting that the term " hamartoma " should be applied only to lesions where there is clear evidence of an underlying

---

EXPLANATION OF PLATES

FIG. 1.—Squamous-cell carcinoma showing a cell-nest and typical squamous cells. H. & E. ×125.
FIG. 2.—Stratification in a squamous-cell carcinoma which resembles a transitional-cell carcinoma of the renal tract. H. & E. ×102.
FIG. 3.—Papillary arrangement of cells in a squamous-cell carcinoma. H. & E ×83.
FIG. 4.—Pseudo-acinar arrangement in a squamous-cell carcinoma. H. & E. ×83.
FIG. 5.—Basal (oat)-cell carcinoma. H. & E. ×77.
FIG. 6.—Rosette and ribbon structure in a basal (oat)-cell carcinoma. H. & E. ×115.
FIG. 7.—Adenocystic carcinoma. Mucicarmine ×77.
FIG. 8.—Epithelial filaments in oedematous stroma giving a pseudo-cartilaginous appearance in an adenocystic carcinoma. H. & E. ×77.
FIG. 9.—Cylindrical structures in an adenocystic carcinoma adjacent to mucous glands showing basal cell hyperplasia. H. & E. ×115.
FIG. 10.—Well-differentiated adenocarcinoma. H. & E. ×77.
FIG. 11.—Syncytial groups of cells in an adenocarcinoma with a well developed fibrous stroma. H. & E. ×115.
FIG. 12.—Adenocarcinoma developing in a bronchial mucous gland. H. & E. ×115.
FIG. 13.—Carcinoid tumour showing a mass of uniform polygonal cells. H. & E. ×345.
FIG. 14.—Oncocytoid cells in a carcinoid tumour. H. & E. ×190.
FIG. 15.—Bronchial tumour with morphological similarity to an intestinal carcinoid. H. & E. ×115.
FIG. 16.—Same tumour as Fig. 13 showing acinar arrangement of cells. H. & E. ×110.
FIG. 17.—Bone with marrow in a bronchial carcinoid tumour. H. & E. ×55.
FIG. 18.—Serous cells in bronchial glands adjacent to a carcinoid tumour which resemble the tumour cells. H. & E. ×185.
FIG. 19.—Similar cells to those seen in Fig. 16 and 17 from the bronchial mucosa adjacent to a tumour of mixed histological type. Mucicarmine ×185.
FIG. 20.—Adenochondroma. H. & E. ×74.

1

2

3

4

Shinton.

5

6

7

8

9

10

11

12

13

14

15

16

17

18

19

20

developmental anomaly. The fact that their presence is not detected until the sixth decade is against their being an embyronic anomaly, and their structure has no similarity to the true hamartomas of infancy.

Origin of some mesenchymal tumours from existing bronchial tissue has been demonstrated by Sutherland, Aylwin and Brewin (1953) and confirmed in a leiomyoma and an osteochondroma of the present series. All of these tumours were however centrally located and contained no epithelial elements, but endobronchial tumours with epithelial elements have been reported (Young, Jones, Hughes, Foley and Fox, 1954). There seems no good reason therefore to further sub-divide the mesenchymal tumours into endobronchial and peripheral neoplasms on the basis of differing histogenesis.

Malignant mesenchymal tumours were originally described in considerable numbers before the appreciation that the "oat-cell sarcomas" were epithelial in nature. The few now found can be regarded as having arisen from their respective connective tissues but cases of benign mesenchymal tumours undergoing malignant change have been reported (Brass, 1942 ; Carsarini and Morone, 1949 ; Lowell and Tuhy, 1949 ; Cavin, Masters and Moody, 1958 ; Feldman, 1958).

*Neurogenic tumours.*—All types have been reported but none were seen in the present series. The subject has been reviewed by Quast (1957) who found neurofibromas to be most frequent, followed by neurosarcoma. Neurilemmoma has been reported, once with malignant change. Chemodectomas have been described by Zeman (1956) and Heppleston (1958).

*Foetal tumours.*—Benign granular myeloblastomas have been reported on rare occasions (Peterson, Soule and Bernatz, 1957 ; Novi, 1958).

"*Mixed*" *tumours.*—In all series of bronchogenic carcinomas, cases with two distinct epithelial types in the same tumour are found. The proportion however depends upon the classification used and has hence varied greatly. True carcinosarcomas where the epithelial and mesenchymal tissue is so intermixed that neither is predominant have occurred as also have carcinoma and sarcoma in different areas of the same tumour (Shinton, 1961).

*Anaplastic tumours.*—Any tumour where the cellular structure is so undifferentiated as not to permit classification into any other type.

*Incidence of histological types*

Using the above classification the 694 tumours of this series were analysed. Where dedifferentiation was present the tumour was regarded as having the characters of the most differentiated tissue present. This also applied where a tissue from the same tumour was available from both biopsy, resection and autopsy. The incidence found is given in Table I.

*Reliability of histological classification.*—In 154 cases where the same tumour was classified from a biopsy, resection or autopsy specimen, discrepancy occurred in 15 (10 per cent). Of these 9 were termed squamous-cell carcinoma on bronchoscopic biopsy and anaplastic at subsequent surgical resection, the remaining 6 being called squamous-cell carcinoma after resection and anaplastic at autopsy.

In a 109 random cases the classification was compared with that given by the pathologist who originally reported on the specimen. Some difference was found in 18 cases (16·5 per cent). Re-examination showed that in all but one instance the discrepancy arose in connection with a squamous-cell carcinoma. Ten had been called anaplastic because of dedifferentiation being present ; two were

TABLE I.—*Incidence of Histological Types*

| | Number | Per cent |
|---|---|---|
| *Epithelial* | | |
| Stratified | | |
| Squamous-cell papilloma | — | — |
| Squamous-cell carcinoma | 387 | 56·0 |
| Basal (oat)-cell carcinoma) | 212 | 30·5 |
| Glandular | | |
| Muco-epidermoid adenoma | — | — |
| Acinar adenoma | — | — |
| Adeno-cystic carcinoma | 5 | 0·7 |
| Adenocarcinoma | 32 | 4·6 |
| Carcinoid tumour | 7 | 1·0 |
| *Mesenchymal* | | |
| Fibroma | — | — |
| Fibrosarcoma | — | — |
| Lipoma | — | — |
| Liposarcoma | — | — |
| Leiomyoma | 1 | 0·1 |
| Leiomyosarcoma | — | — |
| Chondroma | 6 | 0·7 |
| Chondrosarcoma | — | — |
| Angioma | — | — |
| Angiosarcoma | — | — |
| *Neurogenic* | | |
| Neurofibroma | — | — |
| Neurilemmoma | — | — |
| Chemodectoma | — | — |
| *Foetal* | | |
| Myoblastoma | — | — |
| Teratoma | — | — |
| *Mixed* | 13 | 1·9 |
| *Anaplastic* (undifferentiated) | 31 | 4·5 |
| *TOTAL* | 694 | 100·0 |

" mixed " tumours ; and the remainder had been termed " columnar ", " alveolar medullary ", " papillary " or " adenocarcinoma ". One of the latter showed the papilliferous areas illustrated in Fig. 3 and the other showed pseudo-acinar formation (Fig. 4). The remaining tumour was an oat-cell carcinoma with rosettes arranged as acini (Fig. 6) and had been regarded as an adenocarcinoma.

### DISCUSSION

The reported incidence of each histological type of lower respiratory tract tumour has been remarkably variable (Reid and Carr, 1961 ; Shinton, 1961). This has been due mainly to the lack of agreement in terminology and classification. Most differences have been in regard to bronchogenic carcinoma which some pathologists subdivide into many groups on the basis of the cell types present. In the proposed classification described here, these have been limited to squamous-cell, basal-cell, adenocarcinoma, mixed and anaplastic carcinoma. The terms columnar, polygonal, spheroidal, large cell, small cell, round and clear cell carcinoma have been avoided, as have the structural descriptive terms, medullary, trabecular, mucoid, papillary, and pleomorphic, because these could be applied to tumours in each of the three differentiated groups. Alveolar cell carcinoma is considered to be a form of spread, rather than a histological type. The use of these less specific terms has been found to be responsible for some of the differences between pathologists' reports on the same tumour. The main source of difference, however, was over tumours having both differentiated and undifferentiated areas, some classifying them as in this investigation by the most differentiated tissue present, while other regarded them all as undifferentiated carcinomas.

When comparing the incidence of histological types in different series it is important that the source of material be taken into consideration. In the present series there was a much greater proportion of squamous-cell carcinoma specimens obtained by surgical resection than from autopsy. This may have been due to case selection, the autopsy subjects being ones considered inoperable by the clinician. Furthermore, the cases included in the present series represent only half the number clinically diagnosed as having bronchogenic carcinoma, at the particular hospital during the period of study. If the patients, from whom no histological material was available, were considered to be inoperable, then the histological distribution found in the autopsy series represents their occurring in about 60 per cent of cases. This would give an incidence of about 40 per cent each for squamous and basal (oat)-cell tumours and about 10 per cent for anaplastic tumours. The comparatively low incidence of adenocarcinomas in the present series is similar to that in the Annual Cancer Report of the United Birmingham Hospitals in 1954 and so this may be a feature of the geographical area. The small proportion of " mixed tumours " in the present series is no doubt due to the inclusion in this group of only those with two or more differentiated areas. Had serial sections been examined the incidence might have been higher.

Epithelial tumours of low grade malignancy are comparatively rare but their nomenclature remains none the less confused. They have all tended to be grouped under the title " bronchial adenoma " in spite of some showing malignant behaviour. The term adenoma should be restricted to the acinar and muco-epidermoid tumours. These mucous gland tumours demonstrate a gradation of malignancy from the acinar adenoma through the adenocystic carcinoma to the adenocarcinoma, each being however a distinct entity. The carcinoid is a further separate epithelial tumour which is probably an argentaffinoma similar to those occurring in the intestinal tract. This thesis is not difficult to accept when it is appreciated that the respiratory tract develops in the embryo from the ento-dermal layer. Furthermore, patients have been reported with the " carcinoid syndrome " when only a bronchial tumour of this type has been present (Stanford, Davis, Gunter and Hobart, 1958 ; Dockerty, McGoon, Fontana, and Scudamore, 1958 ; Warner and Southren, 1958 ; Schneckloth, McIsaac and Page, 1959 ; Williams and Azzopardi, 1960), and 5-hydroxytryptamine (serotonin) has been extracted from them (Sandler, Scheuer and Watt, 1961 ; Warner, Kirschner and Warner, 1961). The peripheral " tumourlets " described by Prior and Jones (1952) have been regarded as areas of broncho-epithelial proliferation rather than neoplasms. The cubical lining epithelium in some mesenchymal tumours has also been regarded as an epithelial proliferation, here akin to the connective tissue stroma of the epithelial tumours. The term " hamartoma ", sometimes used for this group is better reserved for the true developmental anomalies of the foetus and neonate.

The classification presented here while a compromise on some points is an attempt to provide a logical basis upon which pathologists can agree. Until some better agreement in classification and terminology has been reached it will remain difficult to assess the relationship of histological type to prognosis following the various forms of treatment now available.

SUMMARY

A histological classification of lower respiratory tract tumours has been based upon material removed at bronchoscopy, surgical resection and autopsy from 694 cases. The incidence of each type has been determined and an assessment made of the reliability of this classification. It is proposed that sub-division of " bronchial carcinoma " be limited to squamous-cell carcinoma, basal (oat)-cell carcinoma, adenocarcinoma, mixed and anaplastic (undifferentiated) carcinoma, and that tumours should be classified by the most differentiated tissue present. The term " bronchial adenoma " should include acinar and muco-epidermoid adenomas only ; the adenocystic carcinomas and the carcinoid tumours being distinct types which are sometimes malignant. The term " hamartoma " should be reserved for true developmental anomalies ; mesenchymal tumours, even when epithelial elements are present should be grouped according to the connective tissue present. A plea is made for an agreed classification in order that comparison of biological characteristics and survival times following various forms of treatment can be made by different authors.

I wish to acknowledge the help and encouragement received from Professor J. W. Orr and Dr. G. M. Bonser, also to thank Mr. S. Gaunt, F.I.M.L.T. for the photographs.

### REFERENCES

ADAMS, M. J. T.—(1957) *Thorax*. **12**, 268.
ALBRECHT, M.—(1904) *Verh. dtsch. path. Ges.*, **7**, 153.
BARNARD, W. G.—(1938) *Acta Un. int. Cancr.*, **3**, 213.
BILLROTH, T.—(1859) *Virchows Arch.*, **17**, 357.
BRASS, K.—(1942) *Frankfurt. Z. Path.*, **55**, 525.
BREWER, D. B., BROOKES, V. S. AND VALTERIS, K.—(1953) *Brit. J. Tuberc.*, **47**, 156.
BURFORD, T. H., CARTER, S., FERGUSON, T. B. AND SPJUT, H. J.—(1958) *J. thorac. Surg.*, **36**, 316.
CARSARINI, A. AND MORONE, C.—(1949) *Boll. Soc. med-chir. Pavia*, **63**, 221.
CHIARI, H.—(1883) *Prag. med. Wschr.*, **8**, 497.
CAVIN, E., MASTER, J. H. AND MOODY, J.—(1958) *J. thorac. Surg.*, **35**, 816.
CLAGETT, O. T.—(1960) *Tex. St. J. Med.*, **56**, 838.
CLERF, L. H. AND BUCHER, C. J.—(1942) *Ann. Otol., etc., St. Louis*, **51**, 836.
COLLINS, N. F.—(1958) *Arch. Surg., Chicago*, **77**, 925.
CRAFOORD, C. AND LINDGREN, A. G. H.—(1945) *Acta chir. scand.*, **92**, 481.
DOCKERTY, M. B., MCGOON, D. C., FONTANA, R. S. AND SCUDAMORE, H. H.—(1958) *Med. Clin. N. Amer.*, **42**, 975.
DOLL, R., HILL, A. B. AND KREYBERG, L.—(1957) *Brit. J. Cancer*, **11**, 43.
ENGELBRETH-HOLM, J.—(1945) *Acta chir. scand.*, **90**, 383.
FELDMAN, P. A.—(1958) *Brit. J. Tuberc.*, **51**, 331.
FELLER, A.—(1922) *Virchows Arch.*, **236**, 470.
FEYRTER, F.—(1958) *Dtsch. med. Wschr.*, **83**, 958.
FOSTER-CARTER, A. F.—(1941) *Quart. J. Med.*, **34**, 139.
GARDIOL, D.—(1959) *Oncologia*, **12**, 304.

GEIPEL, P.—(1931) *Frankfurt. Z. Path.*, **42**, 516.
GIFFORD, J. H. AND WADDINGTON, N. K. B.—(1957) *Brit. med. J.*, i, 723.
HAMPERL, H.—(1937) *Virchows Arch.*, **300**, 46.
HARRIS, W. H.—(1943) *Arch. Path.* (*Lab. Med.*) **35**, 85.
HART, C.—(1906) *Z. Krebsforsch.*, **4**, 578.
HELLWEG, G. AND RICKEN, D.—(1957) *Ibid.*, **62**, 133.
HEPPLESTON, A. G.—(1958) *J. Path. Bact.*, **75**, 461.
HOLLEY, S. W.—(1946) *Milit. Surg.*, **99**, 528.
JOBARD, P., VANDOOREN, M. AND ARON, E.—(1959) *Ann. Anat. Path.* (*Paris*), **4** (2) Suppl., 470.
KERNAN, J. D.—(1935) *Ann. Otol., etc., St. Louis*, **44**, 1167.
KIRKLIN, J. W., McDONALD, J. R., CLAGETT, O. T., MOERSCH, H. J. AND GAGE, R. P. (1955) *Surg. Gynec. Obstet.*, **100**, 429.
KREYBERG, L.—(1961) *Brit. J. Cancer*, **15**, 206.
KUNZ, H.—(1937) *Dtsch. Z. Chir.*, **249**, 109.
LANGHANS, T.—(1871) *Virchows Arch.*, **53**, 470.
LOWELL, L. M. AND TUHY, J. E.—(1949) *J. thorac. Surg.*, **18**, 476.
MOLLER, A.—(1933) *Virchows Arch.*, **291**, 478.
NICHOLSON, W. F., FOX, M. AND BRYCE, A. G.—(1957) *Lancet*, i, 296.
NOVI, I—(1958) *Arch. ital. Chir.*, **83**, 333.
OVERHOLT, R. H. AND BOUGAS, J. A—(1956) *J. Amer. med. Ass.*, **161**, 961.
PAUL, F.—(1930) *Mschr. Ohrenheilk.*, **64**, 669.
PETERSON, P. A., SOULE, E. H. AND BERNATZ, P. E.—(1957) *J. thorac. Surg.*, **34**, 95.
PRIOR, J. T. AND JONES, D. B.—(1952) *Ibid.*, **23**, 224.
QUAST, W. H. A.—(1957) *Arch. Chir. Neerl.*, **9**, 25.
RAMSEY, J. H. AND REIMANN, P. L.—(1953) *Amer. J. Path.*, **29**, 339.
RANGER, D., THACKRAY, A. C. AND LUCAS, R. B.—(1956) *Brit. J. Cancer*, **10**, 1.
REID, J. D. AND CARR, A. H—(1961) *Cancer*, **14**, 673.
SANDLER, M., SCHEUER, P. J. AND WATT, P. J—(1961) *Lancet*, ii, 1067.
SCHNECKLOTH, R. E., McISAAC, W. M. AND PAGE, I. H.—(1959) *J. Amer. med. Ass.*, **170**, 1143.
SHINTON, N. K.—(1961) 'The histology evolution and biological characteristics of lower respiratory tract tumours', M.D. thesis, University of Birmingham.
SMETANA, H. F., IVERSON, L. AND SWAN,, L. L.—(1952) *Milit. Surg.*, **111**, 335.
SNIFFEN, R. C., SOUTER, L. AND ROBBINS, L. L.—(1958) *Amer. J. Path.*, **34**, 671.
STANFORD, W. R., DAVIS, J. E., GUNTER, J. U. AND HOBART, S. G.—(1958) *Sth. med. J., Birmingham*, Ala., **51**, 449.
STOUT, A. P.—(1943) *Arch. Path.* (*Lab. Med.*) **35**, 803.
SUTHERLAND, T. W., AYLWIN, J. A. AND BREWIN, E. G.—(1953) *J. Path. Bact.*, **65**, 93.
THOMAS, C. P. T. AND MORGAN, A. D.—(1958) *Thorax*, **13**, 286.
WARNER, R. R. P., KIRSCHNER, P. A. AND WARNER, G. M.—(1961) *J. Amer. med. Ass.*, **178**, 1175.
*Idem* AND SOUTHERN, A. L.—(1958) *Amer. J. Med.*, **24**, 903.
WEINBERGER, M. A., KATZ, S. AND DAVIS, E.—(1955) *J. thorac. Surg.*, **29**, 626.
WEISEL, W., GLICKLICH, M. AND LANDIS, F. B.—(1955) *Arch. Surg., Chicago*, **71**, 128.
WILLIAMS, E. D. AND AZZOPARDI, J. G.—(1960) *Thorax*, **15**, 30.
WILLIS, R. A.—(1958) 'The borderland of embryology and pathology'. London. (Butterworth & Co.).—(1960) 'Pathology of tumours'. London. (Butterworth & Co.).
WOMACK, N. A. AND GRAHAM, E. A.—(1938) *Arch. Path.* (*Lab. Med.*), **26**, 165.
YOUNG, J. M., JONES, E., HUGHES, F. A., FOLEY, F. E. AND FOX, J. R., Jr.—(1954) *J. thorac. Surg.*, **27**, 300.
ZEMAN, M. S.—(1956) *Ann. Otol., etc., St. Louis*, **65**, 960.